# Entomological Reference Collection: 85 years of contributions to public health

**DOI:** 10.11606/s1518-8787.2023057004963

**Published:** 2023-09-14

**Authors:** Mariana de Carvalho Dolci, Fabrício Auad Spina, Maria Anice Mureb Sallum

**Affiliations:** I Universidade de São Paulo Faculdade de Saúde Pública Departamento de Epidemiologia São Paulo SP Brasil Universidade de São Paulo. Faculdade de Saúde Pública. Departamento de Epidemiologia. São Paulo, SP, Brasil.; II Universidade de São Paulo Faculdade de Saúde Pública Programa de Pós-Graduação em Epidemiologia São Paulo SP Brasil Universidade de São Paulo. Faculdade de Saúde Pública. Programa de Pós-Graduação em Epidemiologia. São Paulo, SP, Brasil.

**Keywords:** Entomology, history, Public health, Epidemics, Collection

## Abstract

The Department of Hygiene of the Faculty of Medicine of São Paulo (FMUSP), organized with the support of the Rockefeller Foundation, became the Institute of Hygiene, with the inaugural class taught by Samuel Darling in 1918. The history of Public Health Entomology is mixed with that of the Institute itself, which became the Faculty of Hygiene and Public Health in 1945. Still in the 1930s, Paulo César de Azevedo Antunes and John Lane organized Public Health Entomology within the Medical Parasitology area of the then Institute of Hygiene. During this period, the entomology laboratory came to be recognized for its research in the systematics of hematophagous insects, as well as in the ecology, biology and behavior of vectors. The Entomological Reference Collection (CER) originated naturally from the research of Paulo César Antunes and John Lane and is a national and international heritage covering primary and secondary types of insect species that are of interest to public health. Over the years, it has been consolidated with the efforts of Augusto Ayroza Galvão, Renato Corrêa, José Coutinho, Nelson Cerqueira, Ernesto Rabello, Oswaldo Forattini and others. In its over eighty years of activities, CER has enabled the training of several scientists able to act in programs of surveillance and control of endemic diseases associated with insect vectors throughout Latin America, in addition to training taxonomists focused on insects of interest in Public Health. Researchers from other Brazilian institutes and abroad joined the entomology laboratory because of its importance and the research developed in it. The growing scientific production made it possible for entomological studies developed at the Faculty of Public Health (FSP) to gain international visibility, contributing to the development of disease prevention and epidemic control actions in the country.

## INTRODUCTION

In the early twentieth century, medical Parasitology and Zoology provided subsidies for surveillance problems and were fundamental for understanding some difficulties faced by public health in Brazil. At the São Paulo Institute of Hygiene, the assembly of the so-called Medical Entomology helped professionals of the past to produce the first works that associated mosquitoes as vectors of infectious agents in Brazil and Latin America based on field observations.

The area has been producing knowledge since the 1920s and bets on the innovation of surveillance, control and intervention processes. The principal contributions of FSP professors and their collaborators linked to the Entomological Reference Collection (CER) can be condensed into major themes and show reflections in other Brazilian states.

Basic research done in universities transfers scientific knowledge to innovate health processes and policies. In the past and with few resources, entomologists have worked on describing mechanisms and processes of transmission of vector-mediated infectious agents. These works positively affect public health to this day problems such as deforestation, climate change, global trade, and human movement directly affect the population.

Our contribution to the area is characterized by the demand for entomological studies. Growing because of environmental and climate change, it remains relevant and is increasingly needed because of the socioeconomic impact that mosquito-related diseases represent on society.

This is a study of historiographical nature, understood here as a study and description of history, and our research is affiliated with the History of Sciences. On the subject, Maria Amélia Dantes^1^ explains that, from the 1980s, historians worked with new historiographical standards and began “to raise, in a more systematic way, public and private archives, Brazilian and foreign, in search of the record of scientific practices”.

The author explains that the Brazilian historiographic production in the History of Sciences is fairly new and that only in recent years this research have revealed unpublished documentary collections. This brings up discussions about the situation these documents are in. Previously, it called for a movement to enhance the documentation of Brazilian scientific institutions^1^.

### History

In 1916, the Rockefeller Foundation’s *International Health Board* agreed to organize and maintain for five years what would become Faculty of Medicine of São Paulo’s Hygiene Department. This department became the Institute of Hygiene and the American Samuel Darling taught the inaugural class on April 6, 1918. The history of Public Health Entomology is mixed with that of the Institute itself, which became the Faculty of Hygiene and Public Health in 1945, under the command of Geraldo Horácio de Paula Souza^2^.

In the 1930s, within the Medical Parasitology of the Hygiene Institute, Paulo César de Azevedo Antunes and John Lane dedicated themselves to organizing Public Health Entomology and developing it as a research topic^3,4^. Both Brazil and the State of Sao Paulo suffered from the impact of the epidemics of malaria and yellow fever, besides other endemics associated with hematophagous insects^5^. It was during this period that the entomology laboratory became a center of worldwide recognition both for its research in hematophagous insect systematics and in ecology, biology and vector behavior^6^.

Because of the importance and scope of the research conducted, researchers from other institutes in Brazil and abroad joined the entomology laboratory during its 85 years of uninterrupted activities. Thus, it was possible to train successive generations of specialists whose actions leveraged and expanded the approaches of public health entomology^3,6^.

### The Reference Entomological Collection

After attending the specialization, respectively, at Johns Hopkins University and Cornell University, Antunes and Lane returned to Brazil and both organized and promoted the pioneering Specialization Course in Medical Entomology, in 1949, which had Oswaldo Paulo Forattini as a student^7^.

**Figure 1:**
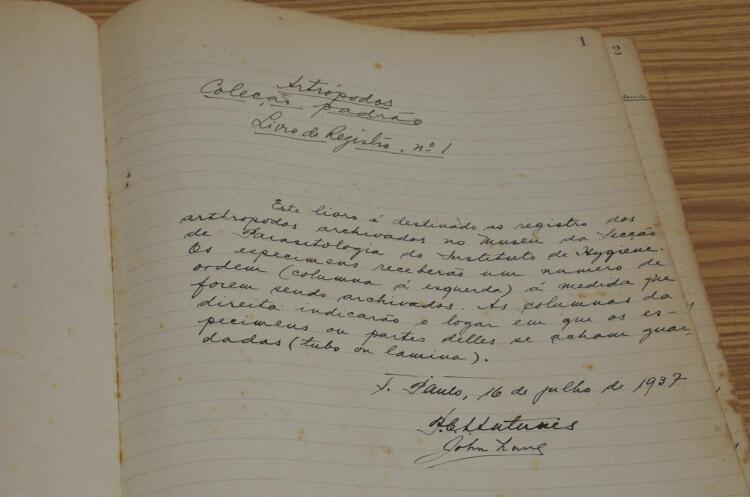
Cover page of the first designated historical book of the Entomological Reference Collection. Paulo César de Azevedo Antunes and John Lane recorded the opening of the Collection on July 16, 1937 [© Marcelo Vigneron]

**Figure 2:**
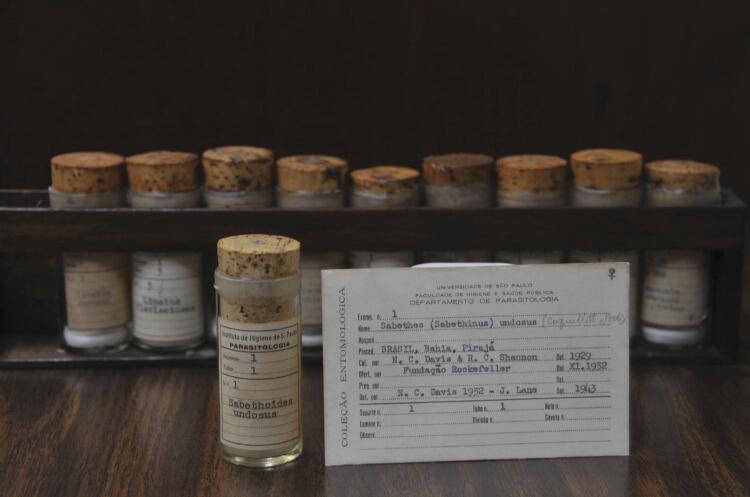
First listed specimen of the Reference Entomological Collection. It is a culicidae specimen of the genus *Sabethes* collected in Pirajá (BA) by Nelson Davis and Raymond Shannon [© Marcelo Vigneron]

Thanks to the encouragement of American entomologists Nelson Davis and Raymond Shannon and the initiatives of Antunes and Lane, on July 16, 1937, the Hygiene Institute witnessed the birth of CER (Figures 1 and 2). The consolidation of the collection as a reference center was attended by Augusto Leopoldo Ayroza Galvão, Renato Corrêa, José de Oliveira Coutinho, Nelson Cerqueira, Ernesto Xavier Rabello, Oswaldo Paulo Forattini and others^6,3^. Forattini’s over six hundred articles on various groups of hematophagous insects, Sallum’s on the family Culicidae and Galati’s on the subfamily Phlebotominae represent the natural continuation and expansion of entomological research begun in the 1930s. In 2020, Galati was included in the list of the world’s most influential researchers in the field of mycology and parasitology^8^. In 2021, Forattini was included and Galati remained on the list^9^.

The CER, as a scientific collection and historical record of insect vectors in the Neotropical Region, is a national and international heritage and contains primary (holotypes and lectotypes) and secondary (paratypes, paralectotypes) types of insect species of public health interest. The collection is continuously enriched with specimens from field capture activities for specific purposes and research developed by teachers and students of public health entomology. In addition, we consider exchanges with Brazilian and foreign institutions, maintained since the creation of the collection. They allow getting samples of species from other geographical regions of the world^3^.

In over eight decades of uninterrupted activities, CER has allowed the training of several hundred entomologists with competence to act in programs of surveillance and control of endemic diseases associated with insect vectors, at the national, international and, in particular, Latin American levels. In addition, we consider the formation of taxonomists with knowledge in several groups of insects of public health importance, especially culicids, sandflies, triatomines, simulids and ceratopogonides.

### Scientific Production

The growing scientific production gave international visibility to the entomological studies developed at the FSP. Among them, it is worth highlighting what showed an association between the emergence of arbovirus epidemics in the urban environment and anthropogenic changes in natural ecosystems. The research theme on the influence of anthropic changes in the natural environment and emergence of infectious diseases mediated by insect vectors was addressed by Paulo Cesar de Azevedo Antunes, John Lane, Oswaldo Paulo Forattini, Almério de Castro Gomes, José Maria Soares Barata, Delsio Natal and Iná Kakitani Murata, in times past^7,3^. These topics remain current and challenging both for health authorities and for the design of entomological surveillance programs for the detection and control of endemic diseases^3^. More recently, the program for sustainable global development recognizes that its success will depend on eradicating extreme poverty in tropical and subtropical countries around the world. In this context, the program includes the need to control endemic diseases, such as malaria, dengue, trypanosomiasis, leishmaniasis, among many other diseases that in most cases affect poor and vulnerable populations^10-12^. The global recognition of the importance of infectious diseases and those mediated by vectors shows the novelty and importance of the work developed by researchers in the entomology laboratory since the 1930s. As an example, it is worth noting Antunes’ pioneering study on aspects of the dynamics of yellow fever transmission and its association with deforestation in Colombia^13^.

Expanding the investigations started by Lane and Antunes, the research developed by Forattini between 1946 and 2009 is recorded in 218 scientific articles, 43 editorials and 14 books on Entomology, Public Health, Epidemiology, Molecular Epidemiology and thoughts on “The Being and Human Being”^14^. Below, it is summarized the chief contributions of the professors of the Entomology Laboratory who, in various ways, contributed to the worldwide recognition of CER as a center for the generation of scientific knowledge. The information was obtained from Sallum et al.^7^ and Sallum^3^, with additional elements.

### Malaria

**Figure 3:**
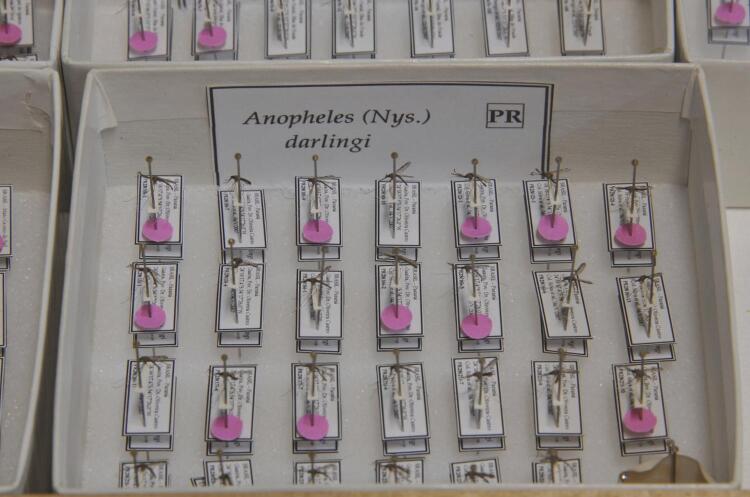
Specimens de *Anopheles darlingi.* Detail of the specimens assembled in an entomological box with their respective location and collector information [© Marcelo Vigneron]

The participation of researchers from the entomology laboratory, especially from Antunes, for the eradication of the mosquito *Anopheles arabiensis* Patton, 1905, from the states of Rio Grande do Norte and Ceará in the 1930s, was important for the success of the program^13^. With the knowledge gained in this activity, it was possible to expand research on malaria vectors and expand activities to other endemic regions of Brazil. Among the studies carried out it is worth mentioning those on the biology and distribution of malaria vectors, bioassays on exposure of *Anopheles darlingi* Root, 1926, to DDT and pyrethrum^3^ (Figure 3). The results of studies conducted by Oliveira Coutinho on the biology and incrimination of *Plasmodium* vector mosquitoes and factors involved in the disease’s transmission guided the campaign to control bromeliad-malaria^3,15^. Studies on this continued with Forattini and Corrêa, from the Environmental Sanitation Superintendence (Susam), and showed that the residual occurrence of the disease in the Atlantic Forest could be explained by the great mobility and behavior of its main vectors, *Anopheles cruzii* Dyar & Knab, 1908, and *Anopheles bellator*, Dyar & Knab,1906^16,17^.

### Visceral and cutaneous leishmaniasis

The first researches of the entomology laboratory on aspects of the epidemiology of cutaneous and visceral leishmaniasis were carried out by Antunes, Galvão and Coutinho, between 1930 and 1940. From the 1950s to the 1980s, Forattini conducted investigations on cutaneous leishmaniasis that allowed the recognition of areas with endemic transmission and the definition of factors involved in the epidemiology of the disease. Research focused on the elucidation of problems concerning transmission, the existence of natural reservoirs of *Leishmania* spp., Phlebotominae systematics, aspects of ecology and behavior of vector species of protozoa. Foci of infection were found in the states of São Paulo, Mato Grosso do Sul, Paraná, Amapá and Rondônia. Founding foci in São Paulo was surprising because, until that time, there was no record of the disease in southern and southeastern states of Brazil. The research allowed pioneering findings on the wild reservoirs of leishmaniasis and the proposal of an epidemiological model of the transmission of cutaneous leishmaniasis and the cycles that parasites could present in nature^3^. The studies were continued by Almério Gomes and Eunice Aparecida Bianchi Galati and expanded knowledge about the biology, behavior and ecology of vectors, besides describing the epidemiological patterns of visceral and cutaneous leishmaniasis in the states of São Paulo, Mato Grosso do Sul and states of the Brazilian Amazon^18^. It is noteworthy that the research conducted by Galati focused both on the Phlebotominae systematics and on the biology, ecology and behavior of its main vectors. As a result, the researcher published 195 original articles, four book chapters, as well as texts and abstracts published in congress annals that she took part in together with her collaborators in Brazil and abroad. The studies on the Phlebotominae system generated a robust body of knowledge about the fauna of the Neotropical Region and Brazil in particular. The proposal to reclassify the subfamily, based on cladistic analysis results, brought unprecedented information about the morphology and added evolutionary knowledge about the group^19^.

### American Trypanosomiasis

Studies led by Forattini on the transmission of American trypanosomiasis and triatomines added knowledge that was important for the control of vector transmission in Brazil. Francisco Cardoso, who in the 1940s served as assistant to the chair of Hygiene, whose title belonged to Paula Souza, had reported that by the first months of 1940 only eight confirmed cases of Chagas disease in São Paulo had been published. Among prophylactic measures, identification and control of triatomines, Cardoso reports a mission of the Hygiene Institute headed by Paula Souza to the municipality of Ituverava (SP) to monitor cases^20^. That same year, Cardoso examined samples of *Triatoma infestans* Klug, 1834, collected by Samuel Pessoa in Itaporanga (SP), confirming the contamination of these by *Trypanosoma cruzi*. This resulted in the communication of new cases of Chagas disease in São Paulo, as well as a warning made by Pessoa regarding the “sanitary importance of this parasitosis in the State of São Paulo”^21^. At the end of the 1940s, Forattini and Oswaldo José da Silva reiterated the warning, reinforcing the need to expand investigations into Chagas disease. This need was proven with the high rate of triatomines contaminated by *T. cruzi* found by Forattini and Silva^22^ ( 1949) in the countryside of the State of São Paulo. With collaborations maintained with researchers from the Secretary of Health of the State of São Paulo, Forattini published a series of 21 articles, known as “Ecological aspects of American trypanosomiasis”, between 1970 and 1980^7^. Based on the knowledge acquired in the research, the Health Department modified the measures adopted to control the endemic disease in the state. Thus, the control went from an action with full insecticide coverage, called the “trawler” phase, to “selective control” according to the evidence of the studies, thus eliminating *T. infestans*, the most important vector. This result served as a model for the proposals for the control of Chagas disease by the Federal Government and later by the Southern Cone countries^3^. The article on the biogeography, origin and distribution of triatomine domiciliation in Brazil revealed the distribution pattern of *Triatoma infestans* in South America^23^. The work is considered by experts as the most important on the subject and was worth highlighting in the book entitled *Vectors of Chagas Disease in Brazil* organized by Cléber Galvão^24^. The studies on triatomines were also the object of the work of Professor José Barata. Among trips made to the regions of Planalto Paulista, Vale do Ribeira and other destinations, Barata could compose a series of articles that dealt with the ecology and morphology of triatomines. He was also among the first researchers to use scanning electron microscopy in his studies, paying special attention to the morphology of the eggs of these hematophagous hemipterans. Working with several entomological groups related to Public Health, Barata left 80 articles and eight book chapters, having been honored with the description of a species of triatomine, the *Triatoma baratai* Carcavallo & Jurberg,2000.^25^

### Ecology, Biology, Behavior and Systematics of Culicidae

Summarizing in a few paragraphs the contributions generated over more than 80 years of activities is a challenging task. At the time of the formation of CER, there were two groups of excellence in public health entomology in Brazil. Manguinhos, Fiocruz (RJ) and the emerging FSP. CER was born as a natural consequence of the research developed by Antunes and Lane^6^. However, research on mosquitoes began before the creation of CER, in 1932, when Paulo Antunes assumed the leadership of the Section of Applied Parasitology and Rural Hygiene. He clearly saw the importance of the knowledge of Medical Entomology, especially in relation to the transmission of malaria and yellow fever^13^.

The culicids involved descriptions of new species, distribution of mosquitoes, determination of the competence and vector capacity of species of the genera *Psorophora, Aedes, Mansonia, Culex* and *Haemagogus* to transmit the yellow fever virus, vertical transmission of the yellow fever virus and vector competence of the urban, *Aedes aegypti* (Linnaeus, 1762) and proof of the vector competence of the species for two strains of the virus that circulated in wild cycles^13^. Taxonomic research was expanded and intensified by Lane and resulted in 151 articles, reviews of various groups, catalogues of species and books on different insect themes and groups. Lane’s work includes two classic books on neotropical Culicids published in 1953^26^,the first catalogue of neotropical Culicids^27^ and the pioneering book on the Sabetines of the Americas, with previously unpublished illustrations by Nelson Cerqueira^28^. The three works are the only ones in this category that address reviews of neglected groups of wild Culicidae^3^.

Forattini dedicated himself to studies on hematophagous insects from 1952 and they were intensified throughout his career as a professor and researcher at FSP. Details about the contributions of Forattini and his collaborators are detailed in Sallum et al.^7^ (2007), Reis et al.^14^ (2016) and Sallum^3^ (2019). Briefly, the research led by Forattini and collaborators addresses ecological aspects within the field of epidemiology knowledge, what is defined as ecological epidemiology^3^. The topics investigated include studies of natural outbreaks of arboviruses and the dynamics of transmission of these infectious agents to humans, considering environmental determinants. Forattini’s collaboration with American researchers resulted in a pioneering study that showed that the *Psorophora ferox* mosquito (Humboldt, 1819) is competent to transmit the Rocio virus in the laboratory. In addition, Forattini and collaborators observed that the household habits of the *Aedes scapularis* mosquito (Rondani, 1848) may explain the species’ participation in the transmission of the Rocio virus in areas of Vale do Ribeira, State of São Paulo. His investigations into mosquitoes intensified in the second half of the 1970s and continued into later years. The several dozen publications show both the originality and importance of the scientific work of Forattini and his collaborators. They addressed aspects of the ecology, epidemiology and systematics of culicidae from Brazil and, in particular, from the State of São Paulo^14,3^. It is worth mentioning the publication of Medical Culicidology in two volumes, with volume 1 being awarded by the *Brazilian Chamber of Books* in 1997^7^.

The research on culicidae was expanded and intensified with the studies developed by Sallum and his collaborators from 1985. The first publications were because of collaborations with Forattini and continued in consecutive years until 2002, with the absence of the professor. After this period, Sallum and collaborators published 157 original articles and four chapters in books on different topics in knowledge of public health entomology. Sallum’s scientific work includes taxonomic studies of diverse groups of Anophelinae and Culicinae, descriptions of new species, reviews, phylogenetic studies with morphological and molecular approach, including the characterization of the mitochondrial genome of multiple species of Anophelinae^29,30^ and the genus *Culex*^31^. Other research approaches by Sallum and collaborators include aspects of the epidemiology and ecology of malaria, anopheline biology, ecology and behavior of culicids, list of species present in remote areas of the Brazilian Amazon, molecular characterization of the yellow fever virus^32^, dengue^33^ and Saint Louis^34^, incrimination of *Haemagogus leucocelaenus* (Dyar, 1925) and *Aedes serratus* (Theobald, 1901) as vectors of the Yellow Fever virus in Rio Grande do Sul in 2008^35^. Investigations into environmental changes that include both degradation and disturbance of the Amazon rainforest and occurrence of malaria are important contributions, as they evidenced factors that act on vector mosquitoes and the occurrence of the disease. The impact of global trade on the occurrence of malaria was evidenced in a study by Chaves^36^. The results of the research should be analyzed considering another investigation led by Chaves and collaborators that showed that the pattern and size of the deforested area are associated with the occurrence of malaria. Thus, the removal of forest cover from an area of 5km^2^ can generate 27 new cases of malaria^37^. In addition, it is worth noting recent articles on the metrics of its transmission in areas of the Brazilian Amazon^2^, the risk of contracting malaria^38^ and the need for additional studies on the fauna of Anophelinae present in the Amazon^39^. The illustrated keys for identifying Anophelinae species from South America^40-42^ are tools developed considering the current needs of malaria control programs in South American countries. Another highlight are the reviews of the Atratus and Educator Groups of *Culex* (*Melanoconion*) that were possible thanks to the use of the CER collection to propose relevant taxonomic changes^43,44^.

Investigations carried out by Mauro Toledo Marrelli resulted in 100 articles that deal with aspects of the dynamics of transmission of bromeliad-malaria in the city of São Paulo, biology, ecology and systematics of culicids, impact of changes in natural landscapes on mosquito populations^45^, environmental changes, vectors and transmission of malaria^46^. The approaches and tools employed by Marrelli allow the recognition of aspects of the phenotype^47^ and genetic structuring of *Aedes albopictus* (Skuse, 1894)^48^ that are associated with environmental changes and processes of selection of insect populations vectors of importance to public health. The recently published articles include discussions on the complexity of malaria elimination in areas outside the Brazilian Amazon^49^.

Closing this list of contributions, still in studies of Culicidae, also stands out Professor Delsio Natal. With over 70 scientific articles about entomology with a focus on public health, 11 book chapters, besides texts published in newspapers and magazines, Professor Delsio dedicated himself mainly to the behavior of vector mosquitoes.

Since the beginning, the FSP incorporates interdisciplinarity as the major approach to its researches. The entomology researches have contributed and continue to contribute to the development of disease prevention and epidemic control actions in the country.
