# Supplementary material for: Entomological Reference Collection: 85 years of contributions to public health
Source: Rev Saude Publica. 2023 Sep 14;57:57. doi: 10.11606/s1518-8787.2023057004963 (PMC10519704; doi:10.11606/s1518-8787.2023057004963)
Supplement: Translation

# Translation: Coleção Entomológica de Referência: 85 anos de contribuições à saúde pública

**Authors:** Mariana de Carvalho Dolci, Fabrício Auad Spina, Maria Anice Mureb Sallum

**Affiliations:** I Brasil Universidade de São Paulo. Faculdade de Saúde Pública. Departamento de Epidemiologia. São Paulo, SP, Brasil.; II Brasil Universidade de São Paulo. Faculdade de Saúde Pública. Programa de Pós-Graduação em Epidemiologia. São Paulo, SP, Brasil.

**Keywords:** Entomologia, história, Saúde Pública, Epidemias, Coleção

## Abstract

O Departamento de Higiene da Faculdade de Medicina de São Paulo (FMUSP), organizado com o apoio da Fundação Rockefeller, tornou-se o Instituto de Higiene, tendo a aula inaugural ministrada por Samuel Darling em 1918. A história da Entomologia de Saúde Pública confunde-se com a do próprio Instituto, que passou a ser a Faculdade de Higiene e Saúde Pública em 1945. Ainda na década de 1930, Paulo César de Azevedo Antunes e John Lane começam a organizar a Entomologia de Saúde Pública dentro da Parasitologia Médica, do então Instituto de Higiene. Durante esse período o laboratório de entomologia passou a ser reconhecido por suas pesquisas em sistemática de insetos hematófagos, bem como na ecologia, biologia e comportamento de vetores. A Coleção Entomológica de Referência (CER) originou-se naturalmente das pesquisas de Paulo César Antunes e John Lane e é um patrimônio nacional e internacional abrangendo tipos primários e secundários de espécies de insetos que apresentam interesse à saúde pública. No decorrer dos anos, consolidou-se com os esforços de Augusto Ayroza Galvão, Renato Corrêa, José Coutinho, Nelson Cerqueira, Ernesto Rabello, Oswaldo Forattini e outros. Em seus mais de oitenta anos de atividades, a CER possibilitou a formação de diversos cientistas aptos a atuar em programas de vigilância e controle de endemias associadas aos insetos vetores em toda a América Latina, além de formar taxonomistas voltados aos insetos de interesse em Saúde Pública. Pesquisadores de outros institutos brasileiros e do exterior juntaram-se ao laboratório de entomologia por conta de sua importância e das pesquisas nele desenvolvidas. A produção científica crescente possibilitou aos estudos entomológicos desenvolvidos na Faculdade de Saúde Pública (FSP) adquirirem uma visibilidade internacional, contribuindo para o desenvolvimento das ações de prevenções de doenças e controle de epidemias no país.

## INTRODUÇÃO

No início do século XX, a Parasitologia e a Zoologia médicas forneceram subsídios para os problemas de vigilância e foram basais para o entendimento de algumas dificuldades enfrentadas pela saúde pública no Brasil. No Instituto de Higiene de São Paulo, a montagem da chamada Entomologia Médica auxiliou os profissionais do passado a produzirem os primeiros trabalhos que associaram os mosquitos como vetores de agentes infecciosos no Brasil e na América Latina, a partir das observações em campo.

A área produz conhecimento desde a década de 1920 e aposta na inovação dos processos de vigilância, de controle e de intervenção. As principais contribuições dos docentes da FSP e de seus colaboradores vinculados à Coleção Entomológica de Referência (CER) podem ser condensadas em grandes temas e evidenciam reflexos em outros estados brasileiros.

As pesquisas básicas feitas em universidades transferem os conhecimentos científicos para inovar processos e políticas de saúde. No passado e com poucos recursos, os entomólogos começaram a trabalhar na descrição de mecanismos e processos de transmissão de agentes infecciosos intermediados por vetores. Esses trabalhos impactam positivamente na saúde pública até os dias de hoje, afinal, problemas como o desmatamento, as alterações climáticas, o comércio global e a movimentação humana atingem diretamente a população.

A nossa contribuição para a área é caracterizada pela demanda por estudos entomológicos. Aumentando em decorrência de mudanças ambientais e climáticas, continua relevante e são cada vez mais necessários por conta do impacto socioeconômico que as doenças relacionadas a mosquitos representam na sociedade.

Esse é um trabalho de natureza historiográfica, entendida aqui como estudo e descrição da História, e nossa pesquisa se filia à História das Ciências. Sobre o tema, Maria Amélia Dantes^1^ nos explica que, a partir da década de 1980, os historiadores passaram a trabalhar com novos padrões historiográficos e começaram “a levantar, de forma mais sistemática, arquivos públicos e privados, brasileiros e estrangeiros, em busca do registro de práticas científicas”.

A autora elucida que a produção historiográfica brasileira na História das Ciências é recente e que só nos últimos anos estas pesquisas têm revelado acervos documentais inéditos. Isso traz à tona discussões sobre a situação em que esses documentos se encontram. Dantes apela para que se faça um movimento de valorização da documentação das instituições científicas brasileiras^1^.

### Histórico

Em 1916, o *International Health Board*, comissão da Fundação Rockefeller, aceitou organizar e manter por cinco anos o que viria a ser o Departamento de Higiene da Faculdade de Medicina de São Paulo. Esse departamento transformou-se no Instituto de Higiene e o americano Samuel Darling ministrou a aula inaugural em 6 de abril de 1918. A história da Entomologia de Saúde Pública confunde-se com a do próprio Instituto, que se tornou a Faculdade de Higiene e Saúde Pública em 1945, sob o comando de Geraldo Horácio de Paula Souza^2^.

Na década de 1930, dentro da Parasitologia Médica do Instituto de Higiene, Paulo César de Azevedo Antunes e John Lane dedicaram-se a organizar a Entomologia de Saúde Pública e a desenvolvê-la como tema de pesquisa^3,4^. Nessa época, tanto o Brasil como o estado de São Paulo sofriam com o impacto das epidemias de malária e febre amarela, além de outras endemias associadas a insetos hematófagos^5^. Foi nesse período que o laboratório de entomologia se tornou centro de reconhecimento mundial tanto por suas pesquisas em sistemática de insetos hematófagos como em ecologia, biologia e comportamento de vetores^6^.

Pela importância e abrangência das pesquisas conduzidas, pesquisadores de outros institutos do Brasil e do exterior juntaram-se ao laboratório de entomologia durante os seus 85 anos de atividades ininterruptas. Dessa maneira, foi possível formar gerações sucessivas de especialistas cujas atuações alavancaram e ampliaram as abordagens da entomologia de saúde pública^3,6^.

### A Coleção Entomológica de Referência

Após cursarem a especialização, respectivamente, na Universidade Johns Hopkins e Universidade de Cornell, Antunes e Lane retornaram ao Brasil e ambos organizaram e promoveram o pioneiro Curso de Especialização em Entomologia Médica, em 1949, que teve Oswaldo Paulo Forattini como aluno^7^.

Graças aos incentivos dos entomólogos norte-americanos Nelson Davis e Raymond Shannon e às iniciativas de Antunes e Lane, em 16 de julho de 1937, o Instituto de Higiene testemunhou o nascimento da CER (Figuras 1 e 2). A consolidação da coleção como centro de referência contou com a participação de Augusto Leopoldo Ayroza Galvão, Renato Corrêa, José de Oliveira Coutinho, Nelson Cerqueira, Ernesto Xavier Rabello, Oswaldo Paulo Forattini e outros^6,3^. As mais de seis centenas de artigos de Forattini sobre diversos grupos de insetos hematófagos, de Sallum sobre a família Culicidae e de Galati sobre a subfamília Phlebotominae representam a continuação natural e a ampliação das pesquisas entomológicas iniciadas na década de 1930. Em 2020, Galati foi incluída na lista de pesquisadores mais influentes do mundo na área de micologia e parasitologia^8^. Em 2021, Forattini foi incluído e Galati permaneceu na lista^9^.

**Figura 1:** Página de rosto do primeiro livro de tombo da Coleção Entomológica de Referência. Paulo César de Azevedo Antunes e John Lane registraram a abertura da Coleção no dia 16 de julho de 1937 [© Marcelo Vigneron]

**Figura 2:** Primeiro exemplar tombado da Coleção Entomológica de Referência. Trata-se de um exemplar de culicídeo do gênero *Sabethes* coletado em Pirajá (BA) por Nelson Davis e Raymond Shannon [© Marcelo Vigneron]

A CER, como acervo científico e registro histórico de insetos vetores da Região Neotropical, é patrimônio nacional e internacional e contém tipos primários (holótipos e lectótipos) e secundários (parátipos, paralectótipos) de espécies de insetos de interesse de saúde pública. O acervo é continuamente enriquecido com espécimes oriundos de atividades de captura de campo para fim específico e pesquisas desenvolvidas por docentes e alunos da entomologia de saúde pública. Acresce considerar os intercâmbios com instituições brasileiras e estrangeiras, mantidos desde a criação da coleção. Eles permitem a obtenção de amostras de espécies de outras regiões geográficas do mundo^3^.

Em mais de oito décadas de atividades ininterruptas, a CER permitiu a formação de várias centenas de entomólogos com competência para atuar em programas de vigilância e controle de endemias associadas aos insetos vetores, nos âmbitos nacional, internacional e, em especial, latino-americano. Acresce considerar a formação de taxonomistas com conhecimentos em diversos grupos de insetos de importância em saúde pública, com destaque para os culicídeos, flebotomíneos, triatomíneos, simulídeos e ceratopogonídeos.

### A Produção Científica

A produção científica crescente deu visibilidade internacional aos estudos entomológicos desenvolvidos na FSP. Dentre eles, vale destacar o que demonstrou a presença de associação entre a emergência de epidemias de arboviroses no ambiente urbano e alterações antrópicas nos ecossistemas naturais. O tema de investigação sobre a influência de alterações antrópicas do ambiente natural e emergência de doenças infecciosas intermediadas por insetos vetores foi abordado por Paulo Cesar de Azevedo Antunes, John Lane, Oswaldo Paulo Forattini, Almério de Castro Gomes, José Maria Soares Barata, Delsio Natal e Iná Kakitani Murata, em tempos passados^7,3^. Esses temas continuam atuais e desafiadores tanto para as autoridades de saúde como para o delineamento de programas de vigilância entomológica para a detecção e para o controle de endemias^3^. Mais recentemente, o programa para o desenvolvimento global sustentável reconhece que o sucesso dele dependerá da erradicação da pobreza extrema nos países tropicais e subtropicais de todo o mundo. Nesse contexto, o programa inclui a necessidade de controle de endemias, como a malária, dengue, tripanossomíases, leishmanioses, entre muitas outras doenças que na maioria dos casos acometem populações pobres e vulneráveis^10^. O reconhecimento global sobre a importância das doenças infecciosas em geral e aquelas intermediadas por vetores, em particular, demonstra o ineditismo e a importância dos trabalhos desenvolvidos por pesquisadores do laboratório de entomologia desde a década de 1930. Como exemplo, vale assinalar o estudo pioneiro de Antunes sobre aspectos da dinâmica de transmissão da febre amarela e sua associação com o desmatamento na Colômbia^13^.

Ampliando as investigações iniciadas por Lane e Antunes, as pesquisas desenvolvidas por Forattini entre 1946 e 2009 estão registradas em 218 artigos científicos, 43 editoriais e 14 livros sobre temas de Entomologia, Saúde Pública, Epidemiologia, Epidemiologia Molecular e pensamentos sobre “O Ser e ser humano”^14^. A seguir, apresentamos resumo das principais contribuições dos docentes do Laboratório de Entomologia que, de diversas maneiras, contribuíram para o reconhecimento mundial da CER como centro de geração de conhecimentos científicos. As informações foram obtidas de Sallum et al.^7^e Sallum^3^, com elementos adicionais.

### Malária

A participação de pesquisadores do laboratório de entomologia, em especial de Antunes, para a erradicação do mosquito *Anopheles arabiensis* Patton, 1905, dos estados do Rio Grande do Norte e Ceará na década de 1930, foi importante para o sucesso do programa^13^. Com os conhecimentos adquiridos nessa atuação, foi possível ampliar as pesquisas sobre os vetores de malária e expandir as atividades para outras regiões endêmicas do Brasil. Dentre os estudos realizados naquela oportunidade, vale assinalar aqueles sobre a biologia e distribuição de vetores de malária, bioensaios sobre exposição de *Anopheles darlingi* Root, 1926, ao DDT e ao piretro^3^(Figura 3). Os resultados dos estudos conduzidos por Oliveira Coutinho sobre a biologia e incriminação de mosquitos vetores de *Plasmodium* e fatores envolvidos na transmissão da doença, orientaram a campanha de controle da bromélia-malária^3,15^. Os estudos sobre esta continuaram com Forattini e Corrêa, da Superintendência de Saneamento Ambiental (Susam), e demonstraram que a ocorrência residual da doença na Mata Atlântica poderia ser explicada pela grande mobilidade e o comportamento dos seus vetores principais, *Anopheles cruzii* Dyar & Knab, 1908, e *Anopheles bellator*, Dyar & Knab, 1906^16,17^.

**Figura 3:** Exemplares de *Anopheles darlingi*. Detalhe dos exemplares montados em caixa entomológica com suas respectivas informações de localidade e coletor [© Marcelo Vigneron]

### Leishmanioses Visceral e Tegumentar

As primeiras pesquisas do laboratório de entomologia sobre aspectos da epidemiologia das leishmanioses tegumentar e visceral foram realizadas por Antunes, Galvão e Coutinho, entre 1930 e 1940. A partir de década de 1950 até a de 1980, Forattini conduziu investigações sobre leishmaniose tegumentar que permitiram o reconhecimento de áreas com transmissão endêmica e a definição de fatores envolvidos na epidemiologia da doença. As pesquisas se concentraram na elucidação de problemas concernentes à transmissão, na existência de reservatórios naturais de *Leishmania* spp., sistemática de Phlebotominae, aspectos da ecologia e comportamento de espécies vetores dos protozoários. Foram encontrados focos de infecção nos estados de São Paulo, Mato Grosso do Sul, Paraná, Amapá e Rondônia. O encontro de foco em São Paulo foi surpreendente pois, até aquela época, não havia registro da doença em estados do sul e sudeste do Brasil. As pesquisas permitiram encontros pioneiros sobre os reservatórios silvestres de leishmanias e a proposta de modelo epidemiológico da transmissão da leishmaniose tegumentar e os ciclos que os parasitas poderiam apresentar na natureza^3^. Os estudos foram continuados por Almério Gomes e Eunice Aparecida Bianchi Galati e ampliaram os conhecimentos sobre a biologia, comportamento e ecologia dos vetores, além de descreverem os padrões epidemiológicos das leishmanioses visceral e tegumentar nos estados de São Paulo, Mato Grosso do Sul e estados da Amazônia brasileira^18^. Vale assinalar que as pesquisas conduzidas por Galati focaram tanto na sistemática de Phlebotominae como na biologia, ecologia e comportamento dos seus principais vetores. Como resultado, a pesquisadora publicou 195 artigos originais, quatro capítulos de livros, além de textos e resumos publicados em anais de congressos que ela participou juntamente com seus colaboradores no Brasil e no exterior. Os estudos sobre a sistemática de Phlebotominae geraram corpo robusto de conhecimentos sobre a fauna da Região Neotropical, em geral e do Brasil, em particular. A proposta de reclassificação da subfamília, baseada em resultados de análises cladísticas, trouxe informações inéditas sobre a morfologia e adicionou conhecimentos evolutivos sobre o grupo^19^.

### Tripanossomíase Americana

Os estudos liderados por Forattini sobre a transmissão da tripanossomíase americana e dos triatomíneos, adicionaram conhecimentos que foram importantes para o controle da transmissão vetorial no Brasil. Francisco Cardoso, que na década de 1940 desempenhava a função de assistente da cadeira de Higiene, cuja titularidade pertencia a Paula Souza, havia relatado que até os primeiros meses de 1940 apenas oito casos confirmados da doença de Chagas em São Paulo haviam sido publicados. Dentre medidas profiláticas, identificação e controle de triatomíneos, Cardoso relata uma missão do Instituto de Higiene chefiada por Paula Souza ao município de Ituverava (SP) para acompanhamento dos casos^20^. Nesse mesmo ano, Cardoso examinou amostras de *Triatoma infestans* Klug, 1834, coletados por Samuel Pessoa em Itaporanga (SP), confirmando a contaminação desses por *Trypanossoma cruzi*. Isso resultou na comunicação de novos casos da doença de Chagas em São Paulo, bem como um alerta feito por Pessoa a respeito da “importância sanitária desta parasitose no Estado de São Paulo”^21^. Ao fim da década de 1940, Forattini e Oswaldo José da Silva reiteravam o alerta, reforçando a necessidade de se ampliarem as investigações sobre a doença de Chagas. Tal necessidade se comprovava com a alta taxa de triatomíneos contaminados por *T. cruzi* encontrados por Forattini e Silva^22^ (1949) no interior do Estado de São Paulo. Com as colaborações mantidas com pesquisadores da Secretária de Saúde do Estado de São Paulo, Forattini publicou uma série de 21 artigos, conhecida como “Aspectos ecológicos da tripanossomíase americana”, entre 1970 e 1980^7^. A partir dos conhecimentos adquiridos nas pesquisas, a Secretaria da Saúde modificou as medidas adotadas no controle da endemia no estado. Assim, o controle passou de uma ação com cobertura total de inseticida, denominada fase de “arrastão”, ao “controle seletivo” segundo as evidências dos estudos, eliminando assim *T. infestans*, o vetor mais importante. Esse resultado serviu de modelo para as propostas de controle da doença de Chagas pelo Governo Federal e posteriormente pelos países do Cone Sul^3^. O artigo sobre a biogeografia, a origem e a distribuição da domiciliação de triatomíneos no Brasil revelou o padrão de distribuição do *Triatoma infestans* na América do Sul^23^. A obra é considerada por especialistas como a mais importante sobre o tema e foi merecedora de destaque no livro intitulado *Vetores da Doença de Chagas no Brasil* organizado por Cléber Galvão^24^. Os estudos sobre triatomíneos eram também objeto de trabalho do professor José Barata. Dentre viagens realizadas às regiões do Planalto Paulista, Vale do Ribeira e outros destinos, Barata pôde compor uma série de artigos que versavam sobre a ecologia e morfologia dos triatomíneos. Figurou também entre os primeiros pesquisadores a utilizar a microscopia eletrônica de varredura em seus estudos, dando especial atenção à morfologia dos ovos desses hemípteros hematófagos. Atuando com diversos grupos entomológicos relacionados à Saúde Pública, Barata deixou 80 artigos e oito capítulos de livros, tendo sido homenageado com a descrição de uma espécie de triatomíneo, o *Triatoma baratai* Carcavallo & Jurberg, 2000.^25^

### Ecologia, Biologia, Comportamento e Sistemática de Culicidae

Resumir em poucos parágrafos as contribuições geradas ao longo de mais de 80 anos de atividades é tarefa desafiadora. Na época da formação da CER, existiam dois grupos de excelência em entomologia de saúde pública no Brasil. O de Manguinhos, Fiocruz (RJ) e o emergente da FSP. A CER nasceu como consequência natural das pesquisas desenvolvidas por Antunes e Lane^6^. Todavia, as pesquisas sobre mosquitos se iniciaram em momento anterior à criação da CER, em 1932, quando Paulo Antunes assumiu a chefia da Seção de Parasitologia Aplicada e Higiene Rural. Ele enxergou claramente a importância que teriam os conhecimentos de Entomologia Médica, principalmente no que se relacionava com a transmissão da malária e da febre amarela^13^.

Os culicídeos envolveram descrições de novas espécies, distribuição de mosquitos, determinação da competência e capacidade vetora de espécies dos gêneros *Psorophora, Aedes, Mansonia, Culex* e *Haemagogus* para transmitir o vírus da febre amarela, transmissão vertical do vírus amarílico e competência vetora do urbano, *Aedes aegypti* (Linnaeus, 1762) e a comprovação da competência vetora da espécie para duas cepas do vírus que circulavam em ciclos silvestres^13^. As pesquisas taxonômicas foram ampliadas e intensificadas por Lane e originaram 151 artigos, revisões de diversos grupos, catálogos de espécies e livros sobre temas e grupos diversos de insetos. A obra de Lane inclui dois livros clássicos sobre os culicídeos neotropicais publicados em 1953^26^, o primeiro catálogo de culicídeos neotropicais^27^e o livro pioneiro sobre os sabetíneos das Américas, com ilustrações inéditas de Nelson Cerqueira^28^. As três obras são as únicas nessa categoria que abordam revisões de grupos negligenciados de culicídeos silvestres^3^.

Forattini dedicou-se aos estudos sobre insetos hematófagos a partir de 1952 e foram intensificados ao longo de sua carreira como professor e pesquisador na FSP. Detalhes sobre as contribuições de Forattini e de seus colaboradores estão detalhados em Sallum et al.^7^ (2007), Reis et al.^14^ (2016) e Sallum^3^ (2019). Resumidamente, as pesquisas lideradas por Forattini e colaboradores abordam aspectos ecológicos dentro do campo do conhecimento da epidemiologia, ou seja, o que se define como epidemiologia ecológica^3^. Os temas investigados incluem estudos de focos naturais de arboviroses e das dinâmicas de transmissão desses agentes infecciosos para o homem, considerando determinantes ambientais. A colaboração de Forattini com pesquisadores americanos resultou em estudo pioneiro que demonstrou que o mosquito *Psorophora ferox* (Humboldt, 1819) é competente para transmitir o vírus Rocio em laboratório. Acresce considerar que Forattini e colaboradores observaram que os hábitos domiciliares do mosquito *Aedes scapularis* (Rondani, 1848) podem explicar a participação da espécie na transmissão do vírus Rocio, em áreas do Vale do Ribeira, Estado de São Paulo. Suas investigações sobre mosquitos se intensificaram na segunda metade da década de 1970 e continuaram nos anos posteriores. As várias dezenas de publicações demonstram tanto a originalidade como a importância da obra científica de Forattini e de seus colaboradores. Elas abordaram aspectos da ecologia, epidemiologia e sistemática de culicídeos do Brasil e, em particular, do Estado de São Paulo^14,3^. Vale assinalar a publicação de Culicidologia Médica em dois volumes, sendo o volume 1 premiado pela *Brazilian Chamber of Books* em 1997^7^.

As pesquisas sobre culicídeos foram ampliadas e intensificadas com os estudos desenvolvidos por Sallum e seus colaboradores a partir de 1985. As primeiras publicações foram decorrentes de colaborações com Forattini e continuaram nos anos consecutivos até 2002, com o afastamento do professor. Após esse período, Sallum e colaboradores publicaram 157 artigos originais e quatro capítulos em livros sobre temas diversos do campo de conhecimento da entomologia de saúde pública. A obra científica de Sallum inclui estudos taxonômicos de grupos diversos de Anophelinae e Culicinae, descrições de novas espécies, revisões, estudos filogenéticos com abordagem morfológica e molecular, incluindo a caraterização do genoma mitocondrial de múltiplas espécies de Anophelinae^29,30^ e do gênero *Culex*^31^. Outras abordagens das pesquisas de Sallum e colaboradores incluem aspectos da epidemiologia e ecologia da malária, biologia de anofelíneos, ecologia e comportamento de culicídeos, lista de espécies presentes em áreas remotas da Amazônia brasileira, caracterização molecular do vírus da febre amarela^32^, dengue^33^ e Saint Louis^34^, incriminação do *Haemagogus leucocelaenus* (Dyar, 1925) e *Aedes serratus* (Theobald, 1901) como vetores do vírus Febre Amarela no Rio Grande do Sul em 2008^35^. As investigações sobre alterações ambientais que incluem tanto a degradação como a perturbação da floresta Amazônica e ocorrência de malária são contribuições que merecem destaque, pois evidenciaram fatores que atuam nos mosquitos vetores e na ocorrência da doença. O impacto do comércio global na ocorrência de malária foi evidenciado em estudo de Chaves^36^. Os resultados da pesquisa devem ser analisados considerando outra investigação liderada por Chaves e colaboradores que demonstrou que o padrão e o tamanho de área desmatada estão associados com a ocorrência de malária. Assim, a remoção da cobertura florestal de uma área de 5 km^2^pode gerar 27 casos novos de malária^37^. Ainda no contexto da doença, vale assinalar artigos recentes sobre as métricas de sua transmissão em áreas da Amazônia brasileira^2^, o risco de contrair malária^38^ e a necessidade de estudos adicionais sobre a fauna de Anophelinae presente na Amazônia^39^. As chaves ilustradas para identificação de espécies de Anophelinae da América do Sul^40^são ferramentas elaboradas considerando-se as necessidades atuais dos programas de controle da malária de países da América do Sul. Outro destaque são as revisões dos Grupos Atratus e Educator de *Culex* (*Melanoconion*) que foi possível graças à utilização do acervo da CER para propor alterações taxonômicas relevantes^43,44^.

As pesquisas de Mauro Toledo Marrelli resultaram em 100 artigos que versam sobre aspectos da dinâmica de transmissão da bromélia-malária na cidade de São Paulo, biologia, ecologia e sistemática de culicídeos, impacto das alterações das paisagens naturais nas populações de mosquitos^45^, mudanças ambientais, vetores e transmissão de malária^46^. As abordagens e as ferramentas empregadas por Marrelli permitem o reconhecimento de aspectos do fenótipo^47^e estruturação genética de *Aedes albopictus* (Skuse, 1894)^48^ que estão associados com as alterações ambientais e processos de seleção de populações de insetos vetores de importância à saúde pública. Os artigos publicados recentemente incluem discussões sobre a complexidade de eliminação da malária em áreas fora da Amazônia brasileira^49^.

E encerrando essa lista de contribuições, ainda no campo de estudos de Culicidae, destaca-se também o professor Delsio Natal. Com mais de 70 artigos científicos sobre o tema da entomologia com foco em saúde pública, 11 capítulos de livros, além de textos veiculados em jornais e revistas, o professor Delsio dedicou-se principalmente ao comportamento dos mosquitos vetores.

A FSP carrega consigo a característica da interdisciplinaridade, muitos de seus profissionais da área da Entomologia contribuíram e continuam contribuindo para o desenvolvimento das ações de prevenções de doenças e controle de epidemias no país.
